# Hospital Costs and Fatality Rates of Traumatic Assaults by Mechanism in the US, 2016-2018

**DOI:** 10.1001/jamanetworkopen.2022.18496

**Published:** 2022-06-24

**Authors:** Luke E. Barry, Grainne E. Crealey, Nga T. Q. Nguyen, Thomas G. Weiser, Sarabeth A. Spitzer, Ciaran O’Neill

**Affiliations:** 1 Centre for Public Health, Queen’s University Belfast, Belfast, United Kingdom; 2 John E. Cairnes School of Business and Economics, National University of Ireland, Galway, Ireland; 3 Faculty of Pharmacy, University of Medicine and Pharmacy at Ho Chi Minh City, Vietnam; 4 Department of Surgery, Stanford University, Stanford, California; 5 Department of Surgery, Brigham and Women’s Hospital, Boston, Massachusetts

## Abstract

**Importance:**

Estimates of the total economic cost of firearm violence are important in drawing attention to this public health issue; however, studies that consider violence more broadly are needed to further the understanding of the extent to which such costs can be avoided.

**Objectives:**

To estimate the association of firearm assaults with US hospital costs and deaths compared with other assault types.

**Design, Setting, and Participants:**

The 2016-2018 US Nationwide Emergency Department Sample and National Inpatient Sample, Healthcare Cost and Utilization Project were used in this cross-sectional study of emergency department (ED) and inpatient admissions for assaults involving a firearm, sharp object, blunt object, or bodily force identified using *International Statistical Classification of Diseases, Tenth Revision, Clinical Modification* codes. Differences in ED and inpatient costs (2020 US dollars) across mechanisms were estimated using ordinary least-squares regression with and without adjustments for year and hospital, patient, and injury characteristics. The Centers for Disease Control and Prevention underlying cause of death data were used to estimate national death rates and hospital case-fatality rates across mechanisms. Cost analysis used a weighted sample. National death rates and hospital case-fatality rates used US resident death certificates, covering 976 million person-years. Hospital case-fatality rates also used nationally weighted ED records covering 2.7 million admissions. Data analysis was conducted from March 1, 2021, to March 31, 2022.

**Exposure:**

The primary exposure was the mechanism used in the assault.

**Main Outcomes and Measures:**

Emergency department and inpatient costs per record. National death rates and hospital case-fatality rates.

**Results:**

Overall, 2.4 million ED visits and 184 040 inpatient admissions for assault were included. Across all mechanisms, the mean age of the population was 32.7 (95% CI, 32.6-32.9) years in the ED and 36.4 (95% CI, 36.2-36.7) years in the inpatient setting; 41.9% (95% CI, 41.2%-42.5%) were female in the ED, and 19.1% (95% CI, 18.6%-19.6%) of inpatients were female. Most assaults recorded in the ED involved publicly insured or uninsured patients and hospitals in the Southern US. Emergency department costs were $678 (95% CI, $657-$699) for bodily force, $861 (95% CI, $813-$910) for blunt object, $996 (95% CI, $925-$1067) for sharp object, and $1388 (95% CI, $1254-$1522) for firearm assaults. Corresponding inpatient costs were $14 702 (95% CI, $14 178-$15 227) for bodily force, $17 906 (95% CI, $16 888-$18 923) for blunt object, $19 265 (95% CI, $18 475-$20 055) for sharp object, and $34 949 (95% CI, $33 654-$36 244) for firearm assaults. National death rates per 100 000 were 0.04 (95% CI, 0.03-0.04) for bodily force, 0.03 (95% CI, 0.03-0.03) for blunt object, 0.54 (95% CI, 0.52-0.55) for sharp object, and 4.40 (95% CI, 4.36-4.44) for firearm assaults. Hospital case fatality rates were 0.01% (95% CI, 0.009%-0.012%) for bodily force, 0.05% (95% CI, 0.04%-0.06%) for blunt object, 1.05% (95% CI, 1.00%-1.09%) for sharp object, and 15.26% (95% CI, 15.04%-15.49%) for firearm assaults. In regression analysis, ED costs for firearm assaults were 59% to 99% higher than costs for nonfirearm assaults, and inpatient costs were 67% to 118% higher.

**Conclusions and Relevance:**

The findings of this study suggest that it may be useful for policies aimed at reducing the costs of firearm violence to consider violence more broadly to understand the extent to which costs can be avoided.

## Key Points

**Question:**

What is the association of firearm assaults with US hospital costs and deaths compared with nonfirearm assaults?

**Findings:**

This cross-sectional study, using 3 years of US hospital admission and national US cause of death data on 2.4 million emergency department visits and 184 040 inpatient admissions for assaults, estimated that firearm assaults had the highest national death rates and hospital case-fatality rates compared with nonfirearm assaults. Initial hospital costs were also higher for firearm assault emergency department visits and inpatient admissions.

**Meaning:**

The findings of this study suggest that it may be beneficial for policies aiming to reduce the costs of firearm violence to consider violence more broadly to understand the extent to which costs can be avoided.

## Teaser

This cross-sectional study compares the hospital costs of various mechanisms of physical assault.

## Introduction

Violence—firearm violence in particular—represents a substantial and increasing public health issue in the US.^1,2^ There were 45 222 firearm deaths in the US in 2020,^3^ and it is a leading cause of death among children and adolescents.^4^ The firearm homicide rate is 25 times higher than the non-US Organization for Economic Cooperation and Development country average.^5^ Limited research activity and a muted policy response have accompanied an increase in lives lost and harmed by firearm violence.^6,7,8^

The wider economic cost of firearm violence, including lost productivity and quality of life, is estimated to be in the billions annually with even greater lifetime costs.^9,10,11^ Although the cost of firearm violence extends beyond immediate trauma and the health care system,^10,12,13^ the cost of inpatient hospitalizations from firearm injuries was estimated to be $6.61 billion from 2006 to 2014—approximately $735 million per year^14^ or $911 million including readmission costs.^15^ A large percentage of this cost (40.8%) involved individuals with governmental insurance.^14^ Costs per initial hospital admission due to firearm injury range from $20 000 to $30 000, with most of the injuries reported as assaults.^16,17,18^

Research into firearm violence often focuses on firearm-related deaths, in part owing to information gaps on nonfatal firearm injuries.^17,18^ Policies to reduce firearm homicides translate into a near equivalent reduction in total homicides,^19^ highlighting their relative lethality compared with other mechanisms, although most people survive a firearm assault.^17^ Similarities in the characteristics of firearm vs nonfirearm assaults as well as fatal vs nonfatal shootings further highlight the role that mechanisms can play in an assault^20,21^; firearms can inflict greater local damage and injure more locations on the body.^4^ Research on the cost of firearm violence, although helpful in drawing attention to the issue, often does not consider relative or opportunity costs. Available evidence on the relative cost of firearms has shown the substantially higher health care costs compared with, for example, stab wounds.^4,11,22^

We focused on a wider range of mechanisms used in assaults to examine the cost of violence in the US more broadly while further contextualizing the cost of firearm violence. We estimated emergency department (ED) and inpatient costs during initial hospital encounters, national death rates (NDRs), and hospital case-fatality rates (HCFRs) due to an assault involving a firearm compared with the other main mechanisms of violent crime (unarmed or bodily force, blunt object, or sharp object) listed by the Federal Bureau of Investigation and broadly corresponding to *International Statistical Classification of Diseases, Tenth Revision–Clinical Modification* (*ICD-10-CM*) codes.^23^ We hypothesized that firearm assaults are associated with significantly higher costs and fatalities compared with nonfirearm assaults.

## Methods

In this cross-sectional study, we used the US Nationwide Emergency Department Sample (NEDS) and National Inpatient Sample (NIS), Healthcare Cost and Utilization Project (HCUP), Agency for Healthcare Research and Quality discharge data from 2016 to 2018 because 2016 was the first full year to use *ICD-10-CM* codes; a full data set documentation has been published.^24,25^ Data analysis was conducted from March 1, 2021, to March 31, 2022. The study conforms with the Strengthening the Reporting of Observational Studies in Epidemiology (STROBE) reporting guideline for cross-sectional research studies and was deemed exempt from ethics approval by the Queen’s University Belfast Faculty Research Ethics Committee/Institutional Review Board because anonymized data were used.

The Federal Bureau of Investigation classifies aggravated assaults, robberies, and murders according to those in which the assailant is armed (firearm, sharp object, or blunt object) and unarmed.^26^ We retrospectively identified records for a firearm (codes X93, X94, X95), sharp object (code X99), blunt object (codes Y00, Y08.0), or bodily force (code Y04) assault from the Centers for Disease Control and Prevention *ICD-10-CM* external cause of injury matrix.^27^ Suicide, unintentional, undetermined, and legal intervention injuries were excluded to provide a more consistent comparison of assailant intent and a more targeted policy discussion. Assaults are the most common intent for hospital-reported firearm injuries.^28^ Records involving more than 1 mechanism were few (<0.5%) and were excluded from the analysis, as were sequelae of injuries.

For costs, we applied the hospital-specific cost-to-charge ratios^29^ and inflated them to 2020 US dollars using the Personal Consumption Expenditures–Hospital Care Index from the Bureau of Economic Analysis.^30^ Descriptive statistics of the patient and hospital characteristics related to each record for which hospital, patient, and cost data were available were calculated according to mechanism. Total cost and cost per record for ED and inpatient admissions were calculated across mechanisms. Emergency department and inpatient costs were log transformed to estimate the proportionate difference in costs across mechanism relative to firearms using ordinary least-squares regression. Models were run with and without adjustment for factors that may be related to health outcomes, the likelihood of being in an assault, or assailant intent.^21^ These factors were patient (age, age-squared, sex, age-adjusted Charlson Comorbidity Index,^31,32^ and insurance status), injury (location of the injury or multiple injuries), hospital (urban-rural location, teaching status, and ownership status) characteristics, and year. Race and ethnicity (reported as Asian or Pacific Islander, Black, Hispanic, American Indian, White, and Other) data were available as a single variable in the NIS and, as exposure to firearm violence in the US varies by race,^33^ we adjusted for this variable when examining inpatient costs; these data are not present in the NEDS. Location of injury was categorized using the injury mortality diagnosis matrix for *ICD-10* codes (extremities, head and neck, spine and upper back, torso, unclassifiable [eg, systemic injuries], unspecified [no recorded location was also coded as unspecified], and multiple [≥2 or more injury locations were recorded]) available from the Centers for Disease Control and Prevention.^34^ For variables with less than 5% missing data, we used a complete-case analysis,^35^ which was the case for inpatient costs and HCFRs. If 5% or more of the data were missing, we examined whether missingness was as good as random; if this was not the case, multiple imputation methods were used (eMethods, eTable 2, and eFigure 3 in the Supplement).

We used the underlying cause of death data from the Centers for Disease Control and Prevention Wide-ranging Online Data for Epidemiologic Research (WONDER) database^36^ to estimate NDRs per 100 000 of the US population from 2016 to 2018 (covering 976 million person-years) across assault mechanisms identified using *ICD-10-CM* codes. To estimate HCFRs, we followed the method used by Kaufman et al^17^: (1) identifying the number of fatal assaults (for hospital settings) in the WONDER database by mechanism, (2) identifying the number of nonfatal initial ED admissions using NEDS (ie, excluding individuals who died during the encounter, and sequelae and subsequent admissions), and (3) calculating the proportion of fatal to total (fatal plus nonfatal) cases. Because assaults involving sports equipment (code Y08.0) were not separable from assaults by other means (codes Y08.8, Y08.9) in the WONDER database, we excluded Y08 codes from our estimates of blunt object assault HCFRs and NDRs. In sensitivity analysis, we reestimated ED and inpatient cost per record, HCFRs, and NDRs when including records of undetermined intent alongside assaults to examine whether potential misclassification of assaults would affect results.^37^ To understand whether the exclusion of sports equipment (code Y08.0) affected NDR and HCFR estimates, we compared ED and inpatient cost per record with and without their inclusion.

### Statistical Analysis

All analyses using HCUP data applied survey weights that cover more than 143 million ED and 35 million inpatient records each year and were conducted using Stata, version 16 (StataCorp LLC). The threshold of significance was set at α = .05 using 2-sided testing.

## Results

From 2016 to 2018 data, 2.8 million ED and 197 320 inpatient records were documented as assaults involving bodily force, blunt objects, sharp objects, or firearms only. This number was reduced to 2.7 million ED records when examining HCFRs and 2.4 million ED and 184 040 inpatient records when examining costs (eFigure 1 and eFigure 2 in the Supplement). Across all mechanisms, the mean age of the population was 32.7 (95% CI, 32.6-32.9) years in the ED and 36.4 (95% CI, 36.2-36.7) years in the inpatient setting, 41.9% (95% CI, 41.2%-42.5%) were female in the ED, 19.1% (95% CI, 18.6%-19.6%) of inpatients were female, and 58.1% (95% CI, 57.5-58.8) were male in the ED and 80.9% (95% CI, 80.4-81.4) were male in the inpatient setting. Most patients had public insurance across all mechanisms; 38.6% (95% CI, 37.4-39.9) of those in the ED and 47.1% (95% CI, 45.8-48.4) of inpatients were in receipt of Medicaid. Table 1 and Table 2 provide further details of patient and hospital characteristics per ED and inpatient records across mechanisms. These characteristics relate to records and visits and not unique patients, so we do not know whether patients had multiple visits during this period.^38^

**Table 1.** Patient and Hospital Characteristics per Emergency Department Record According to the Mechanism Used in an Assault

Characteristic	Mechanism of violent crime			
	Bodily force	Blunt object	Sharp object	Firearm
Age, mean (95% CI), y	32.5 (32.4-32.7)	35.6 (35.3-35.8)	33.2 (33-33.5)	29.2 (28.8-29.6)
Female, % (95% CI)	46.5 (45.9-47)	29.3 (28.5-30.1)	22.8 (22-23.6)	12.5 (11.8-13.2)
Male, % (95% CI)	53.55 (53.02-54.08)	70.63 (69.91-71.53)	77.19 (76.40-77.97)	87.50 (86.79-88.19)
Age-adjusted Charlson Comorbidity Index level, mean (95% CI)	0.36 (0.35-0.37)	0.44 (0.42-0.46)	0.3 (0.29-0.32)	0.31 (0.29-0.33)
Insurance status, % (95% CI)				
None	33.4 (32.3-34.5)	38.5 (36.8-40.3)	42.5 (40.6-44.4)	40.9 (36.8-45.2)
Medicare	7.3 (7.1-7.5)	8.1 (7.8-8.5)	5.2 (4.9-5.5)	3.1 (2.8-3.5)
Medicaid	39.1 (38-40.3)	36.9 (35.2-38.6)	35.4 (33.3-37.5)	38.8 (34.7-43.1)
Private	20.1 (19.4-20.8)	16.5 (15.3-17.7)	16.9 (15.9-18)	17.2 (15.2-19.2)
Hospital teaching status, % (95% CI)				
Metropolitan nonteaching	21.7 (20.1-23.4)	18.2 (16-20.6)	15.4 (13.5-17.5)	9.8 (7.6-12.6)
Metropolitan teaching	63.5 (61.4-65.5)	69 (65.5-72.3)	73.3 (70.3-76.1)	84.1 (80.4-87.2)
Nonmetropolitan^a^	14.8 (13.7-15.9)	12.8 (11.2-14.6)	11.3 (9.9-12.8)	6.1 (4.8-7.7)
Hospital region, % (95% CI)				
Northeast	23.8 (21.6-26.1)	23.8 (18-30.9)	27.5 (22.1-33.7)	13.6 (8.3-21.4)
Midwest	24.1 (22.2-26.2)	21.2 (18.3-24.5)	18.8 (16.1-21.9)	26 (17.3-37.2)
South	43.5 (41.2-45.9)	47.4 (42.5-52.3)	46.5 (41.9-51.2)	55.3 (45.8-64.5)
West	8.6 (7.2-10.1)	7.6 (5.9-9.6)	7.1 (5.1-9.7)	5.1 (2.8-9.1)
Hospital control, % (95% CI)				
Government or private	57.3 (55-59.5)	61.6 (57.3-65.7)	64.3 (60.4-68.1)	71.7 (65.1-77.5)
Government, nonfederal	6.8 (6-7.8)	7 (5.9-8.3)	6.7 (5.2-8.5)	5.7 (4-8)
Private, not-for-profit	19.7 (18-21.4)	17.4 (15.1-19.9)	15.9 (13.7-18.4)	15.1 (10.9-20.5)
Private, investor-owned	7.6 (6.7-8.6)	7 (5.9-8.3)	6.3 (5.3-7.5)	3.7 (2.8-4.9)
Private	8.7 (7.4-10.1)	7 (5.3-9)	6.8 (5.3-8.7)	3.8 (2.4-5.9)
Location of injury, % (95% CI)				
Extremities	15.5 (15.3-15.8)	11.2 (9.2-13.5)	26.8 (26.1-27.5)	41.3 (39.8-42.9)
Head and neck	46.7 (46.3-47.2)	59.6 (58.3-60.9)	22 (21.2-22.8)	9.9 (9.3-10.6)
Multiple	18.2 (17.8-18.5)	19.2 (17.9-20.6)	23.2 (21.7-24.8)	30.3 (28.5-32.1)
Spine and upper back	0.3 (0.2-0.3)	0.1 (0.1-0.1)	0 (0-0)	0.4 (0.3-0.5)
Torso	5.7 (5.5-5.8)	3.5 (3.3-3.7)	15.1 (14.2-16.1)	14.3 (13.6-15)
Unclassifiable	0.6 (0.5-0.7)	0.2 (0.2-0.2)	0.2 (0.2-0.3)	0.1 (0.1-0.2)
Unspecified	13.1 (12.6-13.6)	6.3 (5.8-6.8)	12.7 (10.6-15.1)	3.6 (3.2-4.1)
NEDS records, No.				
Weighted population size (2 385 241)	1 905 053	206 226	183 695	90 267
Sample size (554 356)	441 845	48 313	42 919	21 279
NIS records, weighted population size (184 040)	88 000	16 765	33 275	46 000
Ratio of NEDS to NIS records	22:1	12:1	6:1	2:1

Abbreviations: NEDS, Nationwide Emergency Department Sample; NIS, National Inpatient Sample.

^a^ Nonmetropolitan hospitals were not documented as teaching vs nonteaching in the Healthcare Cost and Utilization Project data because rural teaching hospitals were rare.

**Table 2.** Patient and Hospital Characteristics per Inpatient Record According to the Mechanism Used in an Assault

Characteristic	Mechanism of violent crime			
	Bodily force	Blunt object	Sharp object	Firearm
Age, mean (95% CI), y	39.5 (39.2-39.8)	41.1 (40.6-41.7)	35.3 (34.9-35.6)	29.7 (29.5-30)
Female, % (95% CI)	26.1 (25.4-26.8)	15.1 (13.9-16.3)	13.6 (12.8-14.6)	11 (10.4-11.7)
Male, % (95% CI)	73.91 (73.18-74.63)	84.94 (83.67-86.13)	86.36 (85.45-87.21)	88.97 (88.27-89.63)
Age-adjusted Charlson Comorbidity Index, mean (95% CI)	1.13 (1.1-1.16)	1.08 (1.02-1.15)	0.58 (0.55-0.61)	0.53 (0.51-0.56)
Insurance status, % (95% CI)				
None	23.5 (22.5-24.5)	27.9 (26-30)	31.2 (29.5-33)	27.9 (25.9-30)
Medicare	14.4 (13.8-15)	12 (10.9-13.1)	6.2 (5.7-6.8)	3 (2.7-3.4)
Medicaid	44.5 (43.3-45.6)	46.6 (44.4-48.7)	47.6 (45.8-49.4)	51.8 (49.6-54.1)
Private	17.6 (16.9-18.3)	13.5 (12.3-14.9)	15 (14-15.9)	17.2 (16.2-18.3)
Hospital teaching status, % (95% CI)^a^				
Nonmetropolitan	3.6 (3.3-4)	2.7 (2.2-3.3)	2.8 (2.4-3.2)	1.3 (1-1.7)
Metropolitan, nonteaching	13.6 (12.6-14.6)	9.8 (8.6-11.2)	9.6 (8.3-11.1)	7 (5.4-9)
Metropolitan, teaching	82.8 (81.7-83.9)	87.5 (86-88.9)	87.6 (86.1-89)	91.7 (89.7-93.3)
Hospital region, % (95% CI)				
Northeast	21.3 (19.7-22.9)	18.2 (15.9-20.6)	20.1 (17.8-22.6)	14.3 (11.9-17.1)
Midwest	16.9 (15.6-18.3)	15.8 (13.9-17.9)	12.2 (10.7-13.8)	20 (16.6-24)
South	35.3 (33.4-37.1)	37.3 (34.4-40.3)	37.4 (34.7-40.3)	43.5 (39.4-47.6)
West	26.6 (24.8-28.5)	28.8 (26-31.6)	30.3 (27.6-33.2)	22.2 (19.2-25.5)
Hospital control, % (95% CI)				
Government, nonfederal	19.7 (18.1-21.6)	22.7 (20.1-25.5)	27.7 (24.9-30.6)	25.6 (22.4-29.1)
Private, not-for-profit	69.6 (67.8-71.4)	68.7 (65.8-71.4)	63.1 (60.1-65.9)	68.4 (64.8-71.8)
Private, investor-owned	10.6 (9.8-11.5)	8.7 (7.6-10)	9.3 (8.1-10.6)	6.1 (5.1-7.2)
Location of injury, % (95% CI)				
Extremities	11.3 (10.8-11.8)	7.3 (6.5-8.3)	9.8 (9.1-10.6)	27.4 (26.5-28.4)
Head and neck	45.5 (44.6-46.4)	54.4 (52.7-56)	9.3 (8.6-10)	7.3 (6.8-7.9)
Multiple	23.6 (22.9-24.3)	30.5 (29-32)	47.6 (46.3-48.9)	48 (46.9-49)
Spine and upper back	0.7 (0.6-0.9)	0.4 (0.2-0.7)	0.1 (0.1-0.2)	0.9 (0.7-1.1)
Torso	6.3 (5.9-6.7)	4.3 (3.7-5)	29.6 (28.5-30.7)	15.5 (14.7-16.2)
Unclassifiable	1.6 (1.4-1.8)	0.3 (0.2-0.6)	0.4 (0.3-0.6)	0.1 (0.1-0.2)
Unspecified	11.1 (10.5-11.7)	2.8 (2.3-3.4)	3.1 (2.7-3.6)	0.8 (0.7-1.1)
Race and ethnicity, % (95% CI)^b^				
Asian or Pacific Islander	1.9 (1.6-2.4)	1.8 (1.3-2.5)	1.5 (1.1-2.1)	1.1 (0.9-1.3)
Black	27.3 (26.2-28.5)	29.4 (27.3-31.5)	36.8 (35-38.7)	59.8 (57.5-62)
Hispanic	16.7 (15.7-17.7)	19.3 (17.7-21.1)	22.3 (20.7-23.9)	17.6 (15.9-19.4)
American Indian	2.4 (2-2.8)	4.1 (3.1-5.3)	2.4 (1.9-3.1)	0.5 (0.4-0.7)
White	47.5 (46.1-48.8)	41.2 (39.1-43.4)	31.8 (30.1-33.5)	16.8 (15.6-18.1)
Other	4.3 (3.9-4.8)	4.3 (3.5-5.2)	5.2 (4.5-6)	4.2 (3.5-5.1)
NIS records, No.				
Weighted population size (184 040)	88 000	16 765	33 275	46 000
Sample size (36 808)	17 600	3353	6655	9200

Abbreviation: NIS, National Inpatient Sample.

^a^ Rural hospitals were not documented as teaching vs nonteaching in the Healthcare Cost and Utilization Project data because rural teaching hospitals were rare.

^b^ Race and ethnicity are categorized according to Healthcare Cost and Utilization Project. The exact categories include other without further break down into other component categories.

Of persons presenting to the ED due to assault, the largest proportion of females (NEDS, 47%; NIS, 26%) had experienced bodily force; 13% (NEDS) and 11% (NIS) had been assaulted with firearms. Individuals with assault due to firearms had the lowest mean age (NEDS, 29.2 years; NIS, 29.7 years) with most covered by Medicaid (NEDS, 39%; NIS, 52%) or having no insurance (NEDS, 41%; NIS, 28%). Across all assault mechanisms, most occurred in the Southern part of the US. The most common sites of injuries due to firearm assaults were the extremities (NEDS, 41%; NIS, 27%) and multiple locations (NEDS, 30%; NIS, 48%). In the inpatient setting, most bodily force (48%) and blunt object (41%) assaults involved patients who were White, and most sharp object (37%) and firearm (60%) assaults involved patients who were Black. Firearm assaults had the lowest ratio of ED to inpatient records (2:1); for every firearm assault recorded in the inpatient setting, there were 2 ED records, and bodily force had the highest ratio of ED to inpatient records (22:1).

The Figure and eTable 1 in the Supplement present the total costs from 2016 to 2018 and ED and inpatient costs per record by mechanism, as well as NDRs per 100 000 of the US population and HCFRs as a percentage. Total inpatient costs were highest for firearm assaults ($1.6 billion), approximately $1.7 billion when including ED costs. Although bodily force assaults accounted for the highest total ED ($1.3 billion) and combined total ED and inpatient ($2.6 billion) costs, at the cost per record level, ED and inpatient costs were highest for firearm assaults followed by sharp objects, blunt objects, and bodily force. Cost per record for firearm assaults was higher than nonfirearm assaults, although not to the same extent as fatalities. Emergency department costs were $678 (95% CI, $657-$699) for bodily force, $861 (95% CI, $813-$910) for blunt object, $996 (95% CI, $925-$1067) for sharp object, and $1388 (95% CI, $1254-$1522) for firearm assault. Corresponding inpatient costs were $14 702 (95% CI, $14 178-$15 227) for bodily force, $17 906 (95% CI, $16 888-$18 923) for blunt object, $19 265 (95% CI, $18 475-$20 055) for sharp object, and $34 949 (95% CI, $33 654-$36 244) for firearm assault.

**Figure.**
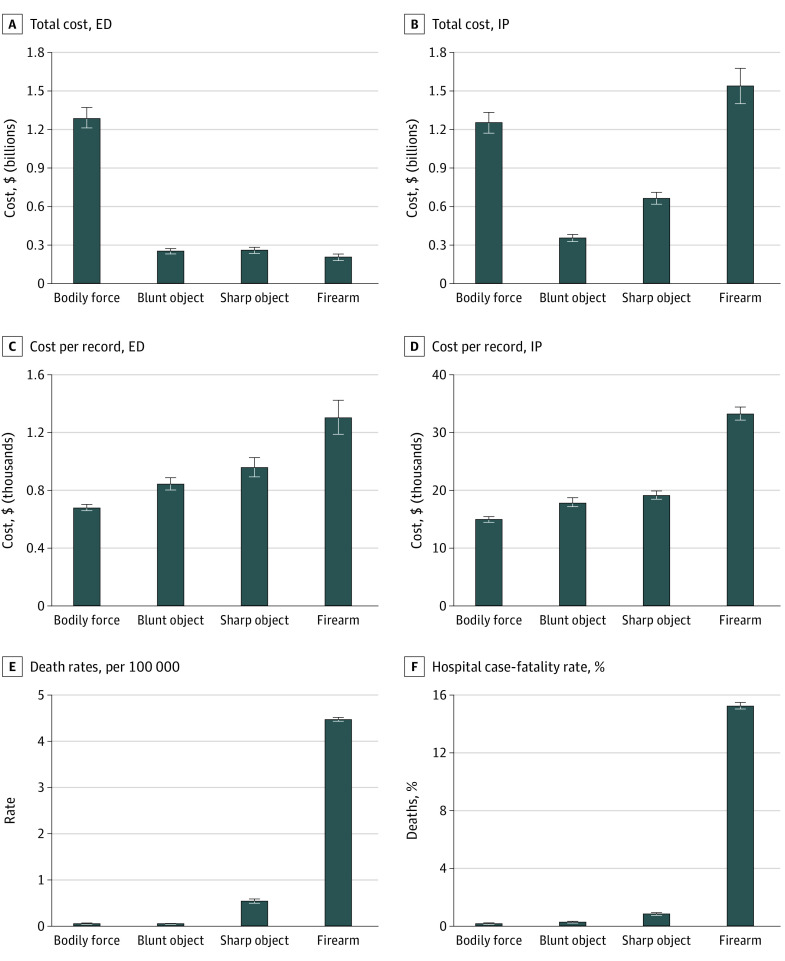
Emergency Department (ED) and Inpatient (IP) Costs and Death Rates According to the Mechanism Used in an Assault All results cover 2016 to 2018. Costs are reported in 2020 USD for total ED (A) and IP (B) costs, cost per record for ED (C) and IP (D) admissions. Death rates (E) are reported per 100 000 US residents, and hospital case fatality rates (F) are reported as the percentage of fatalities in the hospital setting relative to the total number of ED admissions. Both hospital case-fatality rates and national death rates excluded assaults involving sports equipment (*International Statistical Classification of Diseases, Tenth Revision, Clinical Modification* code Y08.0) because these assaults were not separable from assaults by other means (codes Y08.8 and Y08.9) in the Wide-ranging Online Data for Epidemiologic Research database. The estimate of ED records for hospital case-fatality rates excludes subsequent and sequelae admissions and in-hospital deaths but does include records in which cost and patient characteristics were missing.

A similar pattern was evident for NDRs and HCFRs; NDRs per 100 000 were 0.04 (95% CI, 0.03-0.04) for bodily force, 0.03 (95% CI, 0.03-0.03) for blunt object, 0.54 (95% CI, 0.52-0.55) for sharp object, and 4.4 (95% CI, 4.36-4.44) for firearm assaults. The HCFRs were 0.01% (95% CI, 0.009%-0.012%) for bodily force, 0.05% (95% CI, 0.04%-0.06%) for blunt object, 1.05% (95% CI, 1%-1.09%) for sharp object, and 15.3% (95% CI, 15%-15.5%) for firearm assaults. A crude examination of the Figure suggests that HCFRs for firearm assaults were approximately 15 to 1500 times higher than nonfirearm assaults and NDRs were approximately 8 to 110 times higher.

In regression analyses after adjusting for confounders and using a complete-case analysis, ED costs for assaults involving firearms were higher by 99% (95% CI, 85%-114%) vs those for bodily force, 73% (95% CI, 62%-86%) vs blunt object, and 59% (95% CI, 49%-69%) vs sharp object assaults (rescaled from Table 3). Corresponding inpatient costs for firearm assaults were higher by 118% (95% CI, 111%-124%) vs bodily force, 97% (95% CI, 90%-105%) vs blunt objects, and 67% (95% CI, 62%-72%) vs sharp objects (rescaled from Table 4). Multiple imputation results for missing ED costs were not significantly different from the complete-case analysis (Table 3). Results were similar for the unadjusted complete-case analysis of ED and inpatient costs; however, after adjustment, the gradient in costs across mechanisms was more consistent with expectation, ie, bodily force was the least costly, followed by blunt objects, sharp objects, and then firearms.

**Table 3.** Regression Analysis to Estimate the Proportionate Change in ED Assault Costs^a^

Variable	Complete case, % (95% CI)		Adjusted multiple imputation, % (95% CI)	Multiple imputation relative efficiency
	Unadjusted	Adjusted		
Weapon, baseline: firearms				
Bodily force	0.53 (0.49-0.58)	0.50 (0.47-0.54)	0.52 (0.49-0.55)	>0.99
Blunt objects	0.68 (0.63-0.73)	0.58 (0.54-0.62)	0.59 (0.56-0.63)	>0.99
Sharp objects	0.66 (0.62-0.71)	0.63 (0.59-0.67)	0.65 (0.61-0.68)	>0.99
Age, y	NA	1.04 (1.03-1.04)	1.04 (1.03-1.04)	>0.99
Age squared	NA	0.10 (0.10-0.10)	0.10 (0.10-0.10)	>0.99
Female gender	NA	0.90 (0.89-0.91)	0.91 (0.9-0.92)	>0.99
Age-adjusted Charlson Comorbidity Index	NA	1.05 (1.04-1.06)	1.05 (1.04-1.06)	0.99
Hospital control, baseline: government or private				
Government, nonfederal	NA	1.09 (0.98-1.2)	1.07 (0.99-1.16)	>0.99
Private, not-for-profit	NA	1.05 (0.98-1.13)	1.05 (0.98-1.11)	>0.99
Private, investor-owned	NA	0.84 (0.77-0.91)	0.83 (0.77-0.89)	>0.99
Private	NA	1.10 (1.00-1.20)	1.09 (1.01-1.18)	>0.99
Hospital region, baseline: Northeast				
Midwest	NA	0.92 (0.85-0.99)	0.91 (0.84-0.98)	>0.99
South	NA	0.83 (0.76-0.91)	0.83 (0.76-0.9)	>0.99
West	NA	0.99 (0.91-1.07)	0.97 (0.91-1.04)	>0.99
Hospital teaching status, baseline: metropolitan nonteaching				
Metropolitan teaching	NA	1.14 (1.09-1.2)	1.14 (1.09-1.19)	>0.99
Nonmetropolitan	NA	0.99 (0.94-1.03)	0.99 (0.95-1.03)	>0.99
Year, baseline: 2016				
2017	NA	1.08 (1.03-1.13)	1.08 (1.03-1.12)	>0.99
2018	NA	1.05 (0.99-1.10)	1.05 (0.99-1.10)	>0.99
Location of injury, baseline: extremities				
Head and neck	NA	1.59 (1.56-1.62)	1.59 (1.56-1.61)	>0.99
Multiple	NA	1.95 (1.91-1.99)	1.94 (1.90-1.97)	>0.99
Spine and upper back	NA	1.59 (1.50-1.69)	1.57 (1.48-1.68)	>0.99
Torso	NA	1.28 (1.25-1.30)	1.27 (1.25-1.29)	>0.99
Unclassifiable	NA	1.40 (1.33-1.48)	1.39 (1.32-1.46)	0.98
Unspecified	NA	1.11 (1.09-1.13)	1.11 (1.09-1.13)	0.98
Insurance, baseline: no insurance				
Medicare	NA	0.99 (0.96-1.01)	0.99 (0.96-1.01)	>0.99
Medicaid	NA	1.00 (0.97-1.04)	1.00 (0.97-1.03)	>0.99
Private	NA	0.99 (0.97-1.01)	0.99 (0.97-1.01)	>0.99
Intercept	826.67 (753.25-907.25)	304.45 (272.03-340.72)	299.61 (271.19-331.01)	>0.99
Population size, No.	2 385 241	2 385 241	2 805 716	NA
Sample size, No.	554 356	554 356	664 175	NA

Abbreviations: ED, emergency department; NA, not applicable.

^a^The relative efficiency of each variable after 5 imputations is presented. The relative efficiency for all variables is approximately 0.93 or higher, suggesting that 5 imputations were sufficient. Estimates of proportionate changes in costs for assault types vs firearms have been rescaled in the Results section to show the proportionate change in costs for a firearm vs each nonfirearm assault using the following formula ([(1/[coefficient]) – 1] × 100). For example, blunt object assault costs are 58% of firearm assault costs, which equates to firearm assaults being 73% ([(1/0.58) – 1] × 100) higher than a blunt object assault.

**Table 4.** Regression Analysis to Estimate the Proportionate Change in Inpatient Assault Costs^a^

Variable	Complete case, % (95% CI)	
	Unadjusted	Adjusted
Weapon, baseline: firearms		
Bodily force	0.45 (0.43-0.46)	0.46 (0.45-0.47)
Blunt objects	0.54 (0.52-0.56)	0.51 (0.49-0.53)
Sharp objects	0.62 (0.61-0.65)	0.60 (0.58-0.62)
Age, y	NA	1.01 (1.01-1.02)
Age squared	NA	0.99 (0.99-0.99)
Female gender	NA	0.88 (0.86-0.90)
Age-adjusted Charlson Comorbidity Index	NA	1.12 (1.11-1.13)
Hospital control, baseline: government, nonfederal		
Private, not-for-profit	NA	0.95 (0.90-1.01)
Private, investor-owned	NA	0.77 (0.72-0.82)
Hospital region, baseline: Northeast		
Midwest	NA	0.99 (0.93-1.04)
South	NA	1.07 (1.01-1.13)
West	NA	1.40 (1.31-1.50)
Hospital teaching status, baseline: metropolitan nonteaching		
Metropolitan teaching	NA	1.12 (1.05-1.20)
Nonmetropolitan	NA	1.23 (1.17-1.30)
Year, baseline: 2016		
2017	NA	1.01 (0.96-1.06)
2018	NA	1.01 (0.96-1.05)
Location of injury, baseline: extremities		
Head and neck	NA	1.23 (1.20-1.27)
Multiple	NA	1.56 (1.52-1.61)
Spine and upper back	NA	1.35 (1.19-1.54)
Torso	NA	1.21 (1.17-1.26)
Unclassifiable	NA	1.00 (0.90-1.11)
Unspecified	NA	0.90 (0.87-0.94)
Insurance, baseline: no insurance		
Medicare	NA	1.04 (1.00-1.08)
Medicaid	NA	1.11 (1.08-1.14)
Private	NA	1.08 (1.05-1.11)
Race and ethnicity, baseline: White^b^		
Asian or Pacific Islander	NA	1.12 (1.04-1.21)
Black	NA	1.02 (1.00-1.05)
Hispanic	NA	1.04 (1.00-1.07)
American Indian	NA	0.98 (0.90-1.07)
Other	NA	1.04 (0.99-1.10)
Intercept	20 917.64 (20 353.39-21 497.52)	8394.91 (7481.46-9419.90)
Population size, No.	184 040	184 040
Sample size, No.	36 808	36 808

Abbreviation: NA, not applicable.

^a^ Estimates of proportionate changes in costs for assault types vs firearms have been rescaled in the Results section to show the proportionate change in costs for a firearm vs each nonfirearm assault using the following formula ([(1/[coefficient]) – 1] × 100). For example, blunt object assault costs are 51% of firearm assault costs, which equates to firearm assaults being 97% ([(1/0.51) – 1] × 100) higher than a blunt object assault.

^b^ Race and ethnicity are categorized according to Healthcare Cost and Utilization Project. The exact categories include other without further break down into other component categories.

In sensitivity analysis of ED and inpatient cost per record, HCFRs and NDRs were not significantly different when including injuries of undetermined intent alongside assaults except for sharp object HCFRs, which were lower (eTable 1 and eTable 3 in the Supplement). Estimates of ED and inpatient cost per record were not significantly different from each other with and without the inclusion of sports equipment as part of blunt object assaults (eTable 4 in the Supplement).

## Discussion

From 2016 to 2018, bodily force assaults contributed the most to ED costs ($1.3 billion), and firearms contributed the most to inpatient costs ($1.6 billion). Combined ED and inpatient costs were highest for bodily force assaults ($2.6 billion), although this level was attained because bodily force assaults were more prevalent. Firearm assaults had the highest ED and inpatient costs per record, NDR, and HCFR, which were followed by sharp objects, and blunt force assaults (blunt object or bodily force) had the lowest. The highest total inpatient cost and inpatient cost per record for firearm assaults may be explained, in part, by the lower ratio of ED to inpatient records for firearms (2:1) compared with other mechanisms.

Consistent with previous research, most violent injuries recorded in the ED involved males, individuals with public insurance or no insurance, and were recorded in hospitals in the Southern US.^11,16,17,28^ Emergency department records for firearm assaults involved younger patients, on average, and fewer females than nonfirearm assaults. The same pattern was observed for inpatient records in addition to Black patients accounting for the most firearm assaults. The receipt of government insurance (Medicaid) by most individuals involved in assaults highlights the substantial cost borne by the public during initial hospital encounters and the socioeconomic context in which exposure to violence occurs; in particular, exposure to firearm violence for young male individuals who are Black^33^ or those in lower socioeconomic groups.^39^ As with Corso et al,^11^ we noted that greater total health care costs were associated with blunt force injuries (ie, bodily force and blunt objects combined) given their relative abundance. Although a variety of policies may serve to reduce violence in society, measures aimed at firearm violence may highlight a more cost-effective option for policies aiming to reduce morbidity and health care costs per incident.^40^

Emergency department and inpatient cost per record, NDRs and HCFRs were significantly higher when an assault involved a firearm. Using 2016-2017 HCUP data, the Government Accountability Office estimated the cost of initial treatment for a firearm injury to be $1478 for the ED and $30 703 for inpatient care.^18^ Our estimates are similar (ED, $1388; inpatient, $34 949), albeit with differing time periods and data extraction (eg, using *ICD-10-CM* codes, we restricted firearm injuries to those in which the intent was documented as an assault but the Government Accountability Office includes other types of intent such as suicide). Miller and Cohen^22^ estimated the direct medical costs to be $27 299 for a gunshot wound and $16 178 for a stab wound. Although these estimates are older than our data and include costs beyond the initial hospital encounter, the differences are similar to those presented herein for inpatient costs (firearm, $34 949 vs sharp object $19 265). Evidence elsewhere^10,12^ suggests that the initial health care cost following a firearm injury may be a small fraction of the total economic cost and that the gap in costs between firearm and sharp object injuries may widen when considering longer-term productivity losses and mental health care costs.^22^ The younger average age of individuals who experience firearm assaults and their greater likelihood to die helps to explain this widening economic cost, for example, through years of life lost, let alone the humanistic toll on families and communities.^13,41^

In addition to the characteristics of patients involved in assaults, differences in injury characteristics help explain the differences in costs across mechanisms. Bullets may carry considerably more force than other mechanisms and can cause greater damage to local and surrounding structures while also potentially injuring more locations on the body.^4^ We found that assaults involving firearms were more likely to injure multiple locations as well as extremities. In regression analysis, wound location was significantly associated with cost, which may explain the widening cost differential between firearm and nonfirearm assaults after adjusting for confounders. Wound location may also relate to offender intent, ie, if the offender intends to inflict greater damage they may target multiple or specific body parts. Braga and Cook^21^ attempted to control for assailant intent by adjusting for injury location and number of injuries and found that gun caliber was significant in estimating fatality in a firearm assault. This result supports the argument that mechanisms may influence proximal victim outcomes in an assault.

Emergency department and inpatient costs were 59% to 118% higher for firearm vs nonfirearm assaults after adjusting for confounders. We observed an even greater differential between NDRs and HCFRs for firearm compared with nonfirearm assaults. Our estimate of the HCFR for firearms was 15.3% which was lower than the Kaufman et al^17^ estimate of 25%; although these authors examined all settings, for consistency in comparing across mechanisms, we restricted our analysis to the hospital setting. A comparison of HCFRs in the Figure suggests that patients are 15 to 1500 times more likely to die in the hospital due to a firearm assault compared with a nonfirearm assault. Cook,^42^ using ED data, found that individuals who were shot were more than 7 times as likely to die compared with those injured in a knife attack. Our estimate is twice as large as Cook’s estimate because he focused on individuals who were seriously injured in a knife attack. Our results are generally higher than earlier estimates of an individual’s death by a gun (0.41%), knife (0.13%), blunt instrument (0.04%), and unarmed (0.02%) robbery,^43^ although these estimates were older and relate only to robberies and so may not be directly comparable. Our results help to explain the near equivalent reduction in total homicides from policies targeting a reduction in firearm homicides even though substitution may occur in up to 30% of cases^19,44^ (ie, firearm violence may be substituted by nonfirearm violence). Thus, policies targeting a reduction in firearm violence may reduce mortality and, to a lesser extent, health care costs even when substitution with other types of violence occurs. However, as we have shown, the differences in costs between firearm and nonfirearm assaults are still substantial, with firearms being associated with 59% to 118% higher costs.

Stand your ground, right-to-carry, and child access prevention laws appear to be associated with the incidence of firearm deaths^45^ and it is estimated that a strict regime (a combined removal of stand your ground and right-to-carry laws and introduction of child access prevention laws) would reduce firearm homicides by approximately 14% in the US over a 6-year period with an equivalent reduction in total homicides. Assuming a pro rata reduction in firearm assaults, a basic calculation suggests that, even when substitution occurs (approximately 30% of instances) toward the next most costly mechanism in our results (sharp objects), this strict regime may have been associated with a reduction in total combined ED and inpatients costs of approximately $247 million from 2016 to 2018: 14% of the total ED and inpatient costs ($1.7 billion) from initial hospital encounters due to firearm assaults during the same period (calculations in eTable 5 in the Supplement). Although this estimate provides an indication of the potential differences in costs, ours was a cross-sectional analysis, and the presence of each mechanism in an assault would not necessarily be associated with these outcomes. Even so, policies targeting firearm violence may present a cost-effective option in terms of reducing both mortality and health care costs from violence,^40^ especially for individuals of lower socioeconomic status and those of Black race. However, political partisanship may slow progress even when there is broad support across party lines for gun control policies at the mass level.^46^

### Limitations

This study has limitations. With the use of HCUP data to estimate hospital costs and HCFRs according to mechanism, misclassification bias may occur,^18,37^ for example, injuries coded as unintentional or undetermined intent may actually be assaults. It is not possible to know which of these injuries should have been coded as assaults; however, our estimates of ED and inpatient cost per record and HCFRs were similar when reclassifying injuries of undetermined intent as assaults. There may be other classification issues that were beyond the scope of this research, for example, readmissions for an injury may be coded as an initial visit, which would overestimate the count of initial ED encounters. Furthermore, the HCUP includes only costs billed by the facility and not, for example, professional fees,^47^ so our results are likely an underestimation of the initial cost of hospital care. Although the counts of HCUP admissions were nationally representative for inpatients costs and HCFRs, missing ED costs meant that these estimates were not nationally representative, although the results did not differ substantially when imputing ED costs.

## Conclusions

From 2016 to 2018, although firearm assaults accounted for the fewest ED records relative to sharp objects, blunt objects, and bodily force, firearm assaults had the highest ED and inpatient costs per record, NDRs, and HCFRs. The findings of this study suggest that estimates of the total economic cost of firearm violence are useful in drawing attention to this public health issue; however, policies aiming at reducing the cost of firearm assaults should consider violence more broadly to understand the extent to which such firearm assault costs can be avoided.

## Notes

Supplement. **eFigure 1.** Flow Diagram for Retrospective Identification of NEDS Records for Assaults Involving One of Either a Firearm, Sharp Object, Blunt Object, or Bodily Force **eFigure 2.** Flow Diagram for Retrospective Identification of NIS Records for Assaults Involving One of Either a Firearm, Sharp Object, Blunt Object, or Bodily Force **eTable 1.** Total Costs From 2016 to 2018 and Costs per ED and IP Record, Death Rates per 100 000 of the US Population and Hospital Case-Fatality Rates (%) by Assault Mechanism **eMethods.** Missing Data—Multiple Imputation **eTable 2.** Patient, Injury, and Hospital Characteristics by Missing and Nonmissing ED Cost Data With Tests for Differences **eFigure 3.** Trace Plot Showing the Convergence of Imputed ED Costs **eTable 3.** Total Costs From 2016-2018 and Costs per ED and IP Record, Death Rates per 100,000 of the US Population and Hospital Case-Fatality Rates (%) by Assault Mechanism When Including Injuries of Undetermined Intent as Assaults **eTable 4.** 2016-2018 Costs per ED and IP Record by Assault Mechanism With and Without the Inclusion of Sports Equipment (Y08.0) as Part of Blunt Object Assaults (Y00) **eTable 5.** Calculations for Estimating the Expected Reduction in Initial Hospital Costs From a Reduction in Firearm Homicides as a Result of a Strict US Policy Regime Which Introduces Child Access Protection (CAP) Laws and Removes Stand Your Ground (SYG) and Right to Carry (RTC) Laws in the US
eReferences
