# Supplementary material for: Hospital Costs and Fatality Rates of Traumatic Assaults by Mechanism in the US, 2016-2018
Source: JAMA Netw Open. 2022 Jun 24;5(6):e2218496. doi: 10.1001/jamanetworkopen.2022.18496 (PMC9233231; doi:10.1001/jamanetworkopen.2022.18496)
Supplement: Supplement. — eFigure 1. Flow Diagram for Retrospective Identification of NEDS Records for Assaults Involving One of Either a Firearm, Sharp Object, Blunt Object, or Bodily Force eFigure 2. Flow Diagram for Retrospective Identification of NIS Records for Assaults Involving One of Either a Firearm, Sharp Object, Blunt Object, or Bodily Force eTable 1. Total Costs From 2016 to 2018 and Costs per ED and IP Record, Death Rates per 100 000 of the US Population and Hospital Case-Fatality Rates (%) by Assault Mechanism eMethods. Missing Data—Multiple Imputation eTable 2. Patient, Injury, and Hospital Characteristics by Missing and Nonmissing ED Cost Data With Tests for Differences eFigure 3. Trace Plot Showing the Convergence of Imputed ED Costs eTable 3. Total Costs From 2016-2018 and Costs per ED and IP Record, Death Rates per 100,000 of the US Population and Hospital Case-Fatality Rates (%) by Assault Mechanism When Including Injuries of Undetermined Intent as Assaults eTable 4. 2016-2018 Costs per ED and IP Record by Assault Mechanism With and Without the Inclusion of Sports Equipment (Y08.0) as Part of Blunt Object Assaults (Y00) eTable 5. Calculations for Estimating the Expected Reduction in Initial Hospital Costs From a Reduction in Firearm Homicides as a Result of a Strict US Policy Regime Which Introduces Child Access Protection (CAP) Laws and Removes Stand Your Ground (SYG) and Right to Carry (RTC) Laws in the US eReferences [file jamanetwopen-e2218496-s001.pdf]

## Supplementary Online Content

Barry LE, Crealey GE, Nguyen NTQ, Weiser TG, Spitzer SA, O'Neill C. Hospital costs and fatality rates of traumatic assaults by mechanism in the US, 2016-2018. *JAMA Netw Open*. 2022;5(6):e2218496. doi:10.1001/jamanetworkopen.2022.18496

**eFigure 1.** Flow Diagram for Retrospective Identification of NEDS Records for Assaults Involving One of Either a Firearm, Sharp Object, Blunt Object, or Bodily Force

**eFigure 2.** Flow Diagram for Retrospective Identification of NIS Records for Assaults Involving One of Either a Firearm, Sharp Object, Blunt Object, or Bodily Force

**eTable 1.** Total Costs From 2016 to 2018 and Costs per ED and IP Record, Death Rates per 100 000 of the US Population and Hospital Case-Fatality Rates (%) by Assault Mechanism

**eMethods.** Missing Data—Multiple Imputation

**eTable 2.** Patient, Injury, and Hospital Characteristics by Missing and Nonmissing ED Cost Data With Tests for Differences

**eFigure 3.** Trace Plot Showing the Convergence of Imputed ED Costs

**eTable 3.** Total Costs From 2016-2018 and Costs per ED and IP Record, Death Rates per 100,000 of the US Population and Hospital Case-Fatality Rates (%) by Assault Mechanism When Including Injuries of Undetermined Intent as Assaults

**eTable 4.** 2016-2018 Costs per ED and IP Record by Assault Mechanism With and Without the Inclusion of Sports Equipment (Y08.0) as Part of Blunt Object Assaults (Y00)

**eTable 5.** Calculations for Estimating the Expected Reduction in Initial Hospital Costs From a Reduction in Firearm Homicides as a Result of a Strict US Policy Regime Which Introduces Child Access Protection (CAP) Laws and Removes Stand Your Ground (SYG) and Right to Carry (RTC) Laws in the US

**eReferences**

This supplementary material has been provided by the authors to give readers additional information about their work.

© 2022 Barry LE et al. *JAMA Network Open.*

eFigure 1: Flow Diagram for retrospective identification of NEDS records for assaults involving one of either a firearm, sharp object, blunt object, or bodily force.

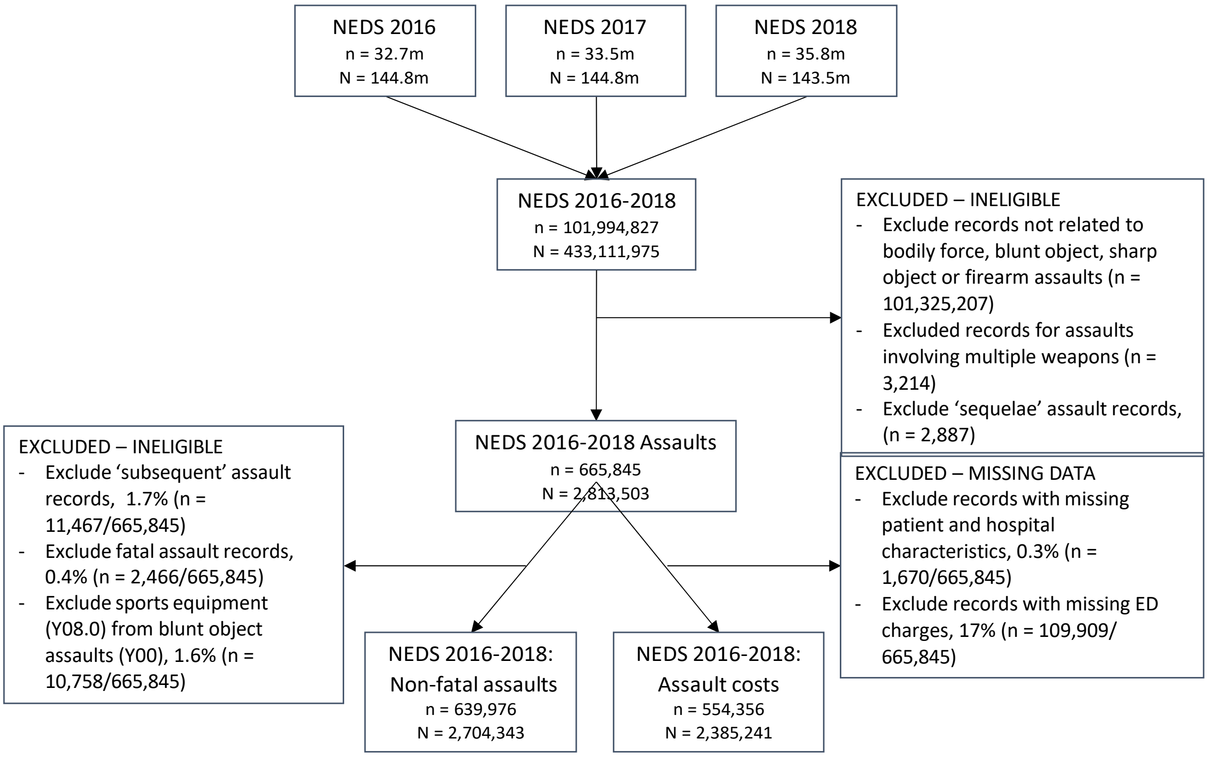


© 2022 Barry LE et al. *JAMA Network Open.*

eFigure 2: Flow Diagram for retrospective identification of NIS records for assaults involving one of either a firearm, sharp object, blunt object or bodily force.

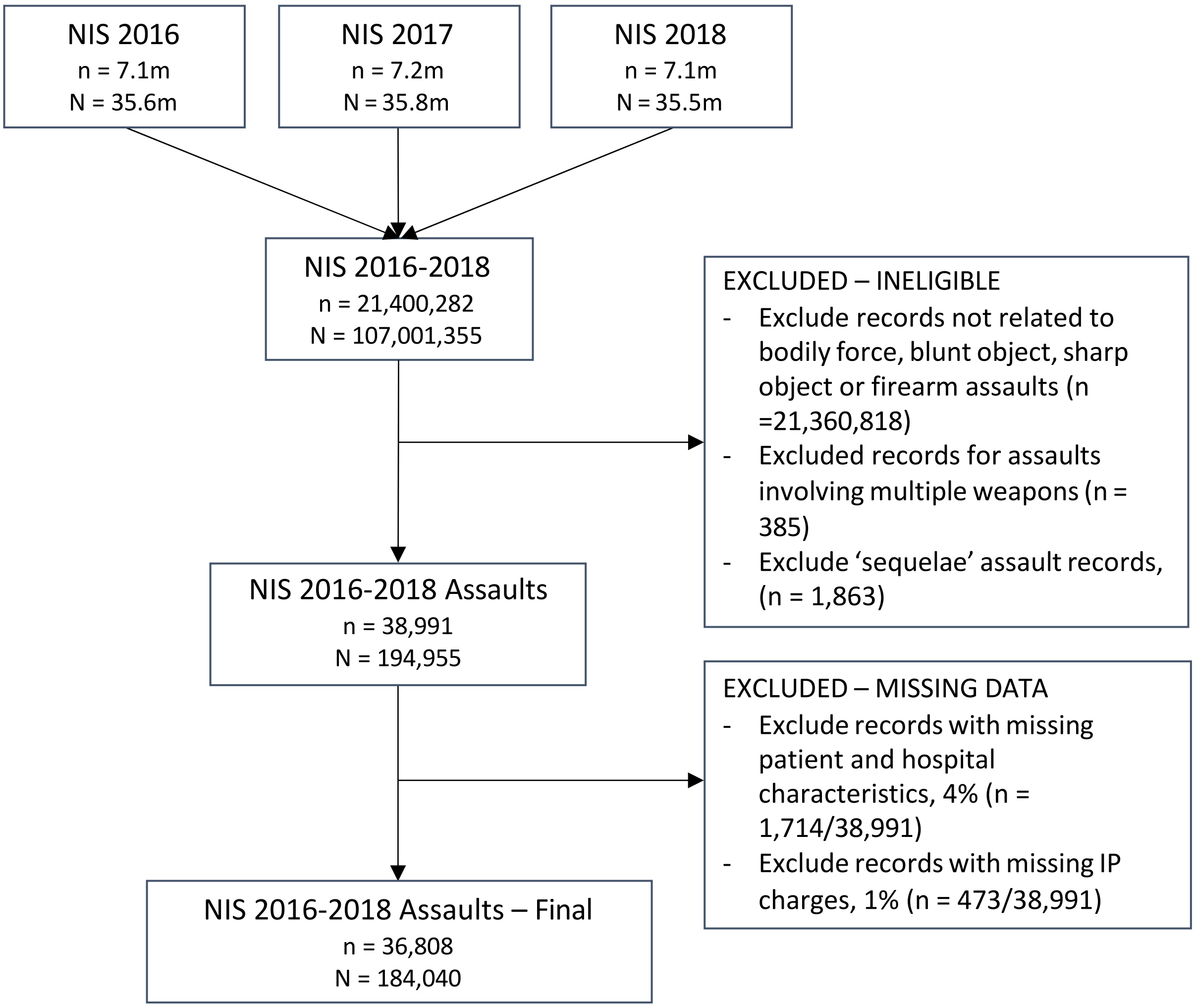


© 2022 Barry LE et al. *JAMA Network Open.*

eTable 1: Total costs from 2016 to 2018 and costs per ED and IP record, death rates per 100 000 of the US population and hospital case-fatality rates (%) by assault mechanism.

| Estimate (95% CI) | **Bodily Force** | **Blunt Object** | **Sharp Object** | **Firearm** |
|---|---|---|---|---|
| Total ED costs | $ 1,292,170,950 (1203860568-1380481333) | $ 177,614,927 (157073504-198156350) | $ 182,887,454 (159280541-206494366) | $ 125,313,477 (99241877-151385078) |
| Cost per ED record | $ 678 (657-699) | $ 861 (813-910) | $ 996 (925-1067) | $ 1,388 (1254-1522) |
| Number of ED records | 1,905,053 | 206,226 | 183,695 | 90,267 |
| | | | | |
| Total IP costs | $ 1,293,810,292 (1207920497-1379700088) | $ 300,192,674 (274641262-325744085) | $ 641,046,088 (592620279-689471896) | $ 1,607,658,898 (1457521241-1757796555) |
| Cost per IP record | $ 14,702 (14178-15227) | $ 17,906 (16888-18923) | $ 19,265 (18475-20055) | $ 34,949 (33654-36244) |
| Number of IP records | 88,000 | 16,765 | 33,275 | 46,000 |
| Death rates per 100,000 US population (excl. Y08) | 0.035 (0.0316 - 0.0391) | 0.029 (0.0257 - 0.0325) | 0.535 (0.52 - 0.549) | 4.397 (4.355 - 4.437) |
| Number of National Deaths | 345 | 284 | 5,220 | 42,915 |
| | | | | |
| Hospital Case-fatality rates (%) (excl. Y08) | 0.0101 (0.0088 - 0.0115) | 0.048 (0.039 - 0.058) | 1.05 (1 - 1.09) | 15.3 (15 - 15.5) |
| Number of Hospital Deaths | 222 | 96 | 2,251 | 18,083 |
| Number of ED records | 2192809 | 198223 | 212928 | 100383 |

Note: Case-fatality rates are estimated within the hospital setting only. Both hospital case-fatality and national death rates exclude assaults involving sports equipment (Y08.0) as these were not separable from assaults by other means (Y08.8, Y08.9) in the WONDER database. The estimate of ED records for case-fatality rates excludes subsequent and sequelae admissions, and in-hospital deaths but includes records where cost and patient characteristics were missing.

© 2022 Barry LE et al. *JAMA Network Open.*

## eMethods. Missing Data - Multiple Imputation

The process that follows refers to the sample of firearm, sharp object, blunt object or bodily force assaults only, not the entire NEDS database (n = 669,620). For variables with missing data accounting for <5% of observations we conducted a complete-case analysis (i.e. used only non-missing observations) as the level of missing data was unlikely to bias the results presented here [1]. eFigure 1 above also outlines this process of exclusion. This was the case for patient age (<0.01% missing), gender (0.01%), insurance status (0.23%). The sample size after excluding these was 664,175. For the NIS data all variables had <5% missingness so only a complete-case analysis was conducted.

For missing ED cost data (17% missing [109,819/664,175]) we first tested for differences in means and counts of variables used in our analysis model (and auxiliary variables for imputation – see below) for those with and without missing ED costs. Chi-squared tests were used to test for differences between categorical variables and t-tests for continuous variables. Comparing variables between those with and without missing cost data allowed us to examine whether data is likely to be missing at random (MAR). If no differences were observed in the analysis variables between missing and complete cost data then missing data were assumed to be MAR and a complete-case analysis was conducted as missing data was deemed unlikely to bias results [1].

© 2022 Barry LE et al. *JAMA Network Open.*

eTable 2: Patient, injury and hospital characteristics by missing and nonmissing ED cost data with tests for differences.

| | Complete ED Cost | Missing ED Cost | p-value |
|---|---|---|---|
| **Age (years), Mean (95% CI)** | 32.7 (32.5-32.9) | 34.6 (34.1-35.1) | <0.001 |
| **Female, % (95% CI)** | 41.9% (41.2-42.5) | 33% (31.5-34.5) | <0.001 |
| **Age-adjusted Charlson Comorbidity Index, Mean (95% CI)** | 0.36 (0.35-0.37) | 0.45 (0.43-0.47) | <0.001 |
| **Insurance Status, % (95% CI)** | | | |
| **No Insurance** | 34.8% (33.7-36.1) | 22.1% (20.1-24.3) | <0.001 |
| **Medicare** | 7.1% (6.9-7.3) | 7.1% (6.5-7.8) | |
| **Medicaid** | 38.6% (37.4-39.9) | 52.4% (49.7-55) | |
| **Private** | 19.4% (18.7-20.1) | 18.4% (16.1-20.8) | |
| **Hospital Teaching Status, % (95% CI)** | | | |
| **Metropolitan non-teaching** | 20.5% (18.9-22.2) | 25.6% (21-30.8) | <0.001 |
| **Metropolitan teaching** | 65.5% (63.4-67.6) | 70.7% (65.3-75.6) | |
| **Non-metropolitan hospital** | 14% (12.9-15.1) | 3.6% (2.4-5.4) | |
| **Hospital Region, % (95% CI)** | | | |
| **Northeast** | 23.7% (21.1-26.5) | 0.3% (0.1-0.5) | <0.001 |
| **Midwest** | 23.6% (21.5-25.7) | 4.6% (3.3-6.4) | |
| **South** | 44.5% (42-47.1) | 1.2% (0.6-2.4) | |
| **West** | 8.2% (6.8-9.9) | 93.9% (91.8-95.5) | |
| **Hospital Control, % (95% CI)** | | | |
| **Government or private** | 58.7% (56.3-61.1) | 45.8% (38.9-52.8) | 0.003 |
| **Government, non-federal** | 6.8% (5.9-7.8) | 13.5% (9.4-19.2) | |
| **Private, not-for-profit** | 19% (17.4-20.7) | 23.2% (18.8-28.3) | |
| **Private, investor-owned** | 7.3% (6.4-8.3) | 7.5% (5.4-10.5) | |
| **Private** | 8.2% (7-9.6) | 10% (6.8-14.5) | |
| **Location of Injury, % (95% CI)** | | | |
| **Extremeties** | 17% (16.7-17.3) | 15.2% (14.8-15.6) | <0.001 |
| **Head & Neck** | 44.5% (44.1-45) | 45.6% (44.8-46.4) | |
| **Multiple** | 19.1% (18.7-19.5) | 21.3% (20.3-22.2) | |
| **Spine and upper back** | 0.2% (0.2-0.3) | 0.2% (0.2-0.3) | |
| **Torso** | 6.5% (6.4-6.7) | 6.9% (6.6-7.2) | |
| **Unclassifiable** | 0.5% (0.4-0.6) | 0.4% (0.4-0.5) | |
| **Unspecified** | 12.1% (11.6-12.6) | 10.4% (9.6-11.3) | |
| **Year, % (95% CI)** | | | |
| **2016** | 33.1% (30.5-35.9) | 31.7% (25.4-38.7) | 0.08 |
| **2017** | 35.7% (33-38.6) | 29.7% (24-36.3) | |
| **2018** | 31.1% (28.9-33.4) | 38.6% (32.2-45.4) | |
| **ED discharge status, % (95% CI)** | | | |
| **Routine** | 89.4% (88.8-90) | 81.2% (79.4-82.8) | <0.001 |
| **Transfer to short-term hospital** | 1.8% (1.7-1.9) | 1.4% (1.2-1.6) | |
| **Transfer (other)** | 1.1% (1-1.2) | 0.9% (0.6-1.1) | |

© 2022 Barry LE et al. *JAMA Network Open.*

| | | | |
|---|---|---|---|
| **Home Health Care (HHC)** | 0.1% (0.1-0.1) | 0% (0-0.1) | |
| **Against medical advice (AMA)** | 2% (1.9-2.2) | 2.8% (2.4-3.2) | |
| **Admitted as an inpatient to this hospital** | 5.1% (4.6-5.7) | 13.4% (11.8-15.2) | |
| **Not admitted to this hospital, discharged alive, destination unknown** | 0.2% (0.2-0.3) | 0.2% (0.1-0.3) | |
| **n = 664,175** | 554,356 | 109,819 | |

© 2022 Barry LE et al. *JAMA Network Open.*

Significant differences were observed for variables with and without missing cost data in NEDS (eTable 2) so multiple imputation was used to impute log of costs. This was conducted using Stata's 'mi' command which applied a Markov Chain Monte Carlo procedure with 5 imputations for missing values. HCUP reports that charge data (which we converted to 2020 costs) is often set to missing because of zero, excessively low or high values [2], these may differ by patient/injury characteristics, year and hospital. It is also recommended that the imputation model be more general than the analysis model, i.e. including covariates used in the analysis model as well as auxiliary variables [1]. As such, our imputation model included the same variables used in our analysis model (patient characteristics [age, age-squared, gender, age-adjusted Charlson comorbidity index, insurance status], injury characteristics [weapon used, location of injury], hospital characteristics [region, teaching status and control], and year) as well as an auxiliary variable: ED discharge status (for the ED cost imputation).

Table 3 present the results of the analytical models with missing cost data imputed. We assume that conditional on the covariates used in our analytical model and our auxiliary variable that data is MAR. The relative efficiency for all variables is approximately 0.96 or higher suggesting that 5 imputations were sufficient. eFigure 3 provides a trace plot which demonstrates good convergence toward a stationary posterior distribution for our imputed variables. Regression coefficients, in particular those for weapon, do not differ significantly from those presented in the complete-case analysis (Table 3)

© 2022 Barry LE et al. *JAMA Network Open.*

eFigure 3: Trace plot showing the convergence of imputed ED costs

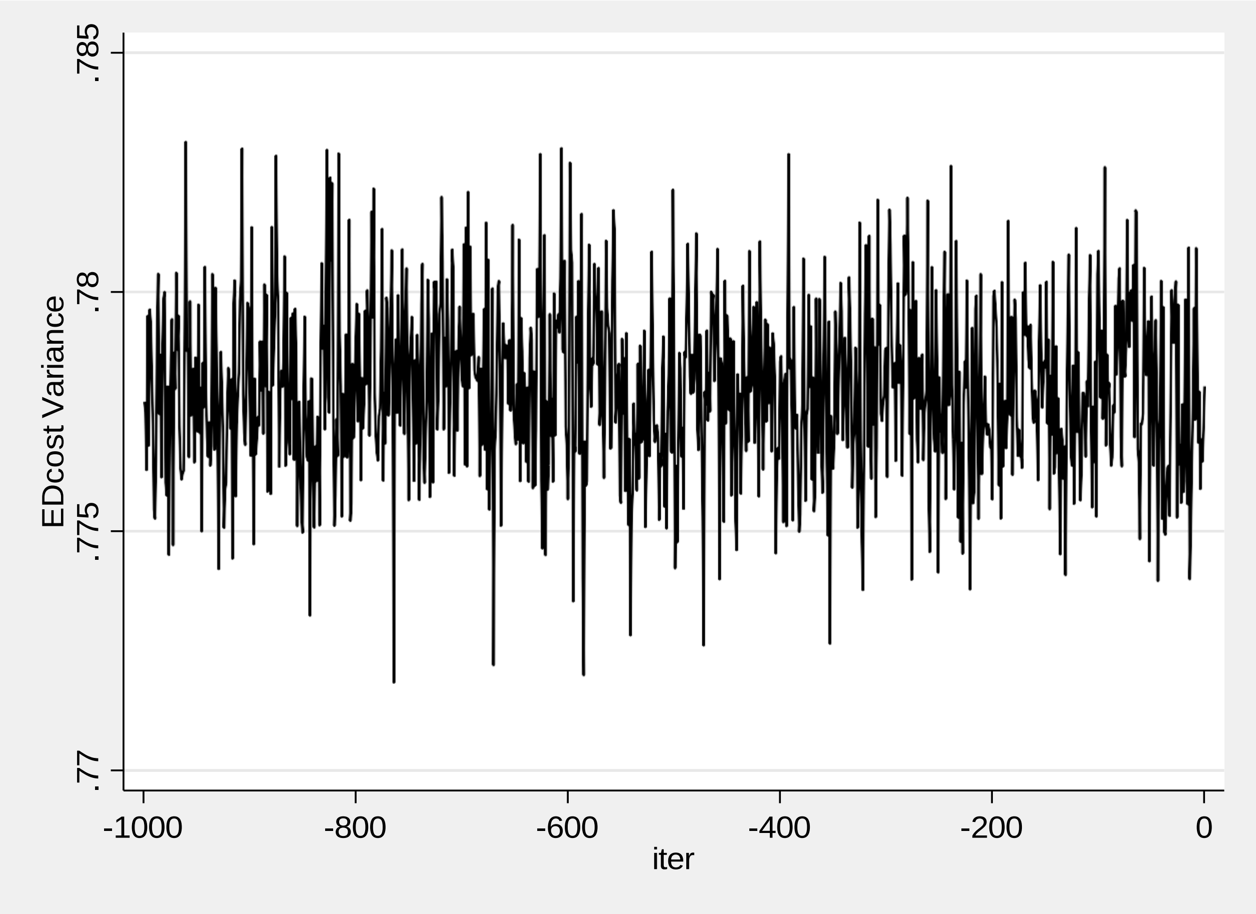


© 2022 Barry LE et al. *JAMA Network Open.*

eTable 3: Total costs from 2016-2018 and costs per ED and IP record, death rates per 100,000 of the US population and hospital case-fatality rates (%) by assault mechanism when including injuries of undetermined intent as assaults.

| Estimate (95% CI) | Bodily Force | Blunt Object | Sharp Object | Firearm |
|---|---|---|---|---|
| **Total ED costs** | $ 1,292,076,759 | $ 184,2898,61 | $ 197,699,256 | $ 134,489,875 (107678305- |
| | (1203771419-1380382099) | (163460445-205119277) | (173487739-221910773) | 161301444) |
| **Cost per ED record** | $ 678 (657-699) | $ 821 (775-866) | $ 885 (819-951) | $ 1373 (1241-1504) |
| **Number of ED records** | 1,904,920 | 224,528 | 223,408 | 97,983 |

| | | | | |
|---|---|---|---|---|
| **Total IP costs** | $ 1,293,810,292 | $ 310,477,509 | $ 671,781,501 | $ 1,721,239,164 |
| | (1214671918-1372948667) | (284843972-336111045) | (621462693-722100310) | (1558667261-1883811068) |
| **Cost per IP record** | $ 14702 (14177-15227) | $ 17686 (16703-18669) | $ 18881 (18110-19652) | $ 34751 (33476-36027) |
| **Number of IP records** | 88,000 | 17,555 | 35,580 | 49,530 |

| | | | | |
|---|---|---|---|---|
| **Death rates per 100,000 US** | 0.0353 (0.0316 - 0.0391) | 0.0299 (0.0265 - 0.0333) | 0.5444 (0.5297 - 0.5590) | 4.4985 (4.4564 - 4.5406) |
| **population (excl. Y08)** | | | | |
| **Number of National Deaths** | 345 | 292 | 5,313 | 43,906 |

| | | | | |
|---|---|---|---|---|
| **Hospital Case-fatality rates (%)** | 0.01 (0.009-0.011) | 0.051 (0.041-0.061) | 0.89 (0.85-0.93) | 14.62 (14.4-14.8) |
| **(excl. Y08)** | | | | |
| **Number of Hospital Deaths** | 222 | 101 | 2,281 | 18,435 |
| **Number of ED records** | 2192660 | 198214 | 254388 | 107694 |

Note: Case-fatality rates are estimated within the hospital setting only. Both hospital case-fatality and national death rates exclude assaults involving sports equipment (Y08.0) as these were not separable from assaults by other means (Y08.8, Y08.9) in the WONDER database. The estimate of ED records for case-fatality rates excludes subsequent and sequelae admissions, and in-hospital deaths but includes records where cost and patient characteristics were missing.

© 2022 Barry LE et al. *JAMA Network Open.*

eTable 4: 2016-2018 costs per ED and IP record by assault mechanism with and without the inclusion of sports equipment (Y08.0) as part of blunt object assaults (Y00). Figures in brackets represent 95% confidence intervals.

| Estimate (95% CI) | Bodily Force | Blunt Object | Sharp Object | Firearm |
|---|---|---|---|---|
| USD Cost/ED Record (incl. Y08) | $ 678 (657-699) | $ 861 (813-910) | $ 996 (925-1067) | $ 1388 (1254-1522) |
| USD Cost/ED Record (excl. Y08) | $ 678 (657-699) | $ 842 (792-892) | $ 994 (924-1065) | $ 1350 (1221-1478) |
| | | | | |
| USD Cost/IP Record (incl. Y08) | $ 14702 (14178-15227) | $ 17906 (16888-18923) | $ 19265 (18475-20055) | $ 34949 (33654-36244) |
| USD Cost/IP Record (excl. Y08) | $ 14753 (14229-15278) | $ 17573 (16513-18632) | $ 19334 (18541-20126) | $ 33492 (32287-34697) |

Note: Small differences in costs for non-blunt objects assaults between those including and excluding Y08 also occur due to changes in the number of assaults involving “Multiple” mechanisms

© 2022 Barry LE et al. *JAMA Network Open.*

eTable 5: Calculations for estimating the expected reduction in initial hospital costs from a reduction in firearm homicides as a result of a strict US policy regime which introduces Child Access Protection (CAP) laws and removes Stand Your Ground (SYG) and Right To Carry (RTC) laws in the US.

| Index | Estimate | 1. Sharp Objects (SO) | 2. Firearms (F) | Calculation |
|---|---|---|---|---|
| a | **National Fatalities** | 5220 | 42,915 | |
| b | **Hospital Fatalities** | 2251 | 18,083 | |
| c | **Hospital Non-fatal Cases** | 212,928 | 100,383 | |
| d | **Hospital Cases** | 215,179 | 118,466 | b + c |
| e | **Hospital Case-Fatality Rate** | 0.010 | 0.153 | b / d |
| f | **Hospital Admission Rate** | 0.431 | 0.421 | b / a |
| g | **Reduction in Fatalities** | | 6,008 | a2 * 0.14 (reduction in firearm homicides from strict regime of 14%) |
| h | **Reduction in Hospital Fatalities** | | 2,532 | f2 * g2 |
| i | **Ratio of fatal to non-fatal admissions** | | 5.55 | c2 / b2 |
| j | **Reduction in F-ED admissions** | | 14,054 | h2 * i2 |
| k | **Reduction in F-IP admissions (2:1)** | | 7,027 | j2 * 0.5 (based on the ratio of ED to IP visits for firearms of 2:1) |
| l | **Hospital admission rate ratio (SO:F)** | 1.023 | | f1 / f2 |
| m | **Increase in SO-ED admissions** | 4,315 | | l1 * j2 * 0.3 (substitution effect of 30%) |
| n | **Increase in SO-IP admissions (6:1)** | 719 | | m1 * 0.167 (based on the ratio of ED to IP visits for sharp objects of 6:1) |
| o | **Cost per ED Admission** | 996 | 1388 | |
| p | **Cost per IP Admission** | 19265 | 34949 | |
| q | **Cost of total ED Admissions** | 4,295,778 | 19,509,993 | (m1 * o1) & (j2 * o2) |
| r | **Cost of total IP Admissions** | 13,853,974 | 245,581,432 | (n1 * p1) & (k2 * p2) |
| s | **Cost of Total Admissions** | 18,149,752 | 265,091,425 | q + r |
| t | **Total offset w/ Substitution** | | 246,941,673 | s2 - s1 |
| u | **Total F Costs (ED & IP)** | | 1,732,972,375 | |
| v | **Total Offset (%)** | | 14% | t2 / u2 |

© 2022 Barry LE et al. *JAMA Network Open.*

eReferences

1. Jakobsen, J.C., et al., *When and how should multiple imputation be used for handling missing data in randomised clinical trials – a practical guide with flowcharts.* BMC Medical Research Methodology, 2017. **17**(1): p. 162.
2. Healthcare Cost and Utilisation Project (HCUP). *NEDS Description of Data Elements*. 2021 [cited 2021 April]; Available from: https://www.hcup-us.ahrq.gov/db/vars/totchg_ed/nedsnote.jsp#general.

© 2022 Barry LE et al. *JAMA Network Open.*
